# Godless owls, devout larks: Religiosity and conscientiousness are associated with morning preference and (partly) explain its effects on life satisfaction

**DOI:** 10.1371/journal.pone.0284787

**Published:** 2023-05-24

**Authors:** Joanna Gorgol, Paweł Łowicki, Maciej Stolarski

**Affiliations:** Faculty of Psychology, University of Warsaw, Warsaw, Poland; Jiangsu University, CHINA

## Abstract

The associations between morningness-eveningness, conscientiousness, and religiosity have not been investigated to date. The aim of the present research was to provide evidence for the relationships between these dimensions. Moreover, we tested whether the well-established link between morningness and life satisfaction could be explained by elevated religiosity of morning-oriented individuals and whether this relationship may be mediated by conscientiousness. The investigation was conducted on two independent samples of Polish adults (*N* = 500 and *N* = 728). Our results corroborated earlier findings that morningness was positively associated with both conscientiousness and satisfaction with life. We also found evidence for a significant positive association between morningness and religiosity. Moreover, controlling for age and gender, we obtained significant mediation effects showing that the association between morningness-eveningness and satisfaction with life might stem, at least in part, from the higher religiosity among morning-oriented individuals, also when conscientiousness was included in the model. It means that more morning-oriented individuals may benefit from higher psychological well-being thanks to both personality characteristics and attitudes towards religion.

## Introduction

One of the most commonly cited Polish proverbs says “kto rano wstaje, temu Pan Bóg daje” [the one who gets up early, gets rewarded by God]. Although its meaning is roughly reflected in popular sayings like “the early bird catches the worm” and “early to bed and early to rise makes a man healthy, wealthy, and wise", the Polish adage adds another dimension to its English-language counterparts as it seeks the source of morning lark’s prosperity in the divine blessing. In the present paper, we aimed to put the folk wisdom expressed in the Polish saying into an empirical test. Thus, across two high-powered studies, we investigated the interplay between morningness-eveningness, conscientiousness, religiosity, and satisfaction with life.

Chronotype, often referred to as morningness-eveningness preference, is a central aspect of individual differences in the area of circadian rhythms [1]. It is manifested in a diurnal preference for the usual timing of one’s sleep-wake cycle and their preferred time for physical and mental activity; some individuals, often referred to as ‘larks’ or Morning-types, prefer going to bed and waking up early, while others, commonly labeled ‘owls’ or Evening-types, prefer later hours. However, most of the population is intermediate in this regard.

Multiple studies show that morningness-eveningness is associated not only with the timing of sleep or waking up but also with many other physical and psychological features, including cognitive effectiveness [2], emotional functioning [3], emotional intelligence [4], or personality [5]. Importantly, chronotype has also marked consequences for human well-being and flourishing. Namely, research indicates that morningness is associated with higher satisfaction with life, construed as a subjective evaluation of the quality of life-based on personal criteria set by individuals [6–8].

The beneficial impact of morningness may rely on several mechanisms. For instance, morningness is associated with better sleep quality and longer sleep duration, which translates into higher life satisfaction [9]. Furthermore, morningness contributes to higher educational achievement [10] which may further translate into life satisfaction [11]. Finally and most notably, prior work established that morningness is also associated with higher scores on a socially desirable personality trait of conscientiousness [12] and that the positive relationship between morning chronotype and well-being is partly mediated through conscientiousness [13]. Conscientiousness, meanwhile, has been established as a robust predictor of life satisfaction and mental health [14].

Although it seems that larks’ conscientiousness may directly contribute to their overall well-being, taking inspiration from the Polish common saying, we suggest that this link may also partly work through higher religious devotion of morning-oriented individuals. This line of reasoning is supported by a number of previous empirical findings.

First, religiosity is positively correlated with conscientiousness and agreeableness [15] and longitudinal research has established that levels of conscientiousness in adolescence predict higher religiosity in early adulthood [16]. Similarly, it has been found that changes in conscientiousness and Eysenckian psychoticism predicted the endorsement of religious values among Australian adolescents [17].

Second, a large body of evidence indicates a generally positive relationship between religiosity and various aspects of psychological well-being, including (but not limited to) happiness [18], optimism [19] or life satisfaction [20]. In addition, research shows religion can replenish and foster self-regulatory strength [21], which, in turn, positively contributes to satisfaction with life [22].

Last but not least, while not yet conclusively established, there is some indirect evidence suggesting that morningness could be positively related to belief in God and religiosity. To start with, a link between morningness and religiosity could be expected because various religions explicitly enjoin healthy sleep patterns and praise those who commit themselves to wake up early [23]. In terms of previous empirical findings, cross-cultural evidence indicates that countries with higher levels of religiousness (e.g., India, Colombia or Iran) show general preference for morningness [24–26]. Moreover, it has been found that morningness on an individual level predicts more conservative moral foundations [27], which are characteristic of religious believers as well [28]. On the other hand, both morningness and religiosity are also inversely related to socially undesirable personality traits commonly known as the Dark Triad, i.e., psychopathy, Machiavellianism, and narcissism [29, 30].

To sum up, morningness has been previously linked with higher rates of life satisfaction and this relationship may partly result from the increased conscientiousness of morning-oriented individuals. Furthermore, considering the nexus between conscientiousness, religiosity, and well-being there are reasons to believe that religiosity could serve as yet another mediator of the relationship between chronotype and satisfaction in life.

### Current research

The main aim of the present research was twofold. First, we wished to replicate findings on the mediating role of conscientiousness in the association between morningness-eveningness and satisfaction with life. Second, we wanted to significantly extend the previous research by examining the incremental validity of an additional mediator, i.e., religiosity. Therefore, we hypothesized that: (1) morningness is associated with higher conscientiousness, (2) morningness is associated with higher religiosity, (3) there is a positive association between religiosity and satisfaction with life. Finally, based on previous research indicating that religiosity is strongly associated with conscientiousness, we tested whether (4) religiosity mediates the association between morningness-eveningness and satisfaction with life and whether (5) this relationship is, in turn, mediated by conscientiousness. To verify the stability and robustness of the conducted analyses, we repeated this investigation in two independent samples.

## Study 1

### Method

#### Participants

A total of 500 participants (302 women and 198 men) aged 18 to 71 years (M = 38.36, SD = 13.93) took part in the first study. All of the participants declared that Poland was their country of origin.

All participants gave written informed consent before participating in the study. The research was conducted in line with the ethical standards of the Declaration of Helsinki, and the procedure was accepted by the Research Ethics Committee at the Faculty of Psychogy, University of Warsaw.

#### Measures

*Chronotype* was measured with the Composite Scale of Morningness (CSM) [31] in a Polish adaptation [32]. The CSM contains 13 items referring to various aspects of circadian functioning (e.g., ‘At what time in the evening do you feel tired and, as a result, in need of sleep?’). Each item is rated on a four or five-point scale. Higher scores indicate greater morningness, while lower scores indicate greater eveningness. Internal consistency of the Polish adaptation of CSM measured with Cronbach’s α amounts to .84 [32], showing high reliability of this scale.

*Belief in God* was measured using a questionnaire consisting of three questions (‘I believe in God’; ‘I believe in a divine being who is involved in my life’; ‘There is no god or high power in the universe’) scored with an 8-point Likert scale from 1 (‘strongly disagree’) to 8 (‘strongly agree’). The total score in this questionnaire is a sum of all the items comprising the scale; hence, the variable ranges from 3 to 24. The same items have been previously used in several empirical investigations [30, 33]. Overall, the measure shows high internal consistency and good construct validity, as evidenced by strong correlations with other religious belief scales [33].

*Life satisfaction* was measured with the Satisfaction with Life Scale (SWLS) [34] in a Polish adaptation [35]. The SWLS contains five items (e.g., ‘I am satisfied with my life’) scored on a 7-point Likert scale ranging from 1 (’completely disagree’) to 7 (’completely agree’). Higher scores indicate greater satisfaction with life. Internal consistency of the Polish adaptation of SWLS measured with Cronbach’s α amounts to .86 [35], showing high reliability of this scale.

*The Big Five personality traits* were measured with the International Personality Item Pool Big Five Factor Markers questionnaire (IPIP-BFM-50) [36] in a Polish adaptation [37] The IPIP-BFM-50 contains 50 items (e.g., ‘Am the life of the party’) scored on a 5-point Likert scale ranging from 1 (’very inaccurate) to 5 (’very accurate). The measure comprises five subscales: extraversion, agreeableness, conscientiousness, emotional stability, and intellect. Internal consistencies of the subscales of the Polish adaptation of IPIP-BFM-50 measured with Cronbach’s α range between .73 and.91 [37], showing sufficient reliability of this scale. Note that in our studies, we focused only on conscientiousness, as it is most strongly correlated with both chronotype [12] and belief in God [15].

#### Statistical analyses

The matrix of partial correlations between the measured variables, as well as the reliability of the psychometric measures and descriptive statistics (means, standard deviations, skewness, kurtosis, and interquartile range), are provided in Table 1.

**Table 1:** Means, standard deviations, skewness, kurtosis, interquartile range, Cronbach’s alphas, and bivariate correlations between variables included in the study 1 (N = 500).

	M	SD	Skewness	Kurtosis	IQR	α	1.	2.	3.	4.
1. Morningness-eveningness	36.05	7.70	-.16	-.55	11	.88	-			
2. Belief in God	16.95	6.55	-.55	-.89	11	.64	.17**	-		
3. Satisfaction with life	20.08	6.40	-.09	-.50	9	.88	.12**	.15**	-	
4. Conscientiousness	3.52	.68	-.18	-.39	10	.81	.25**	.26**	.20**	-

Note. High morningness-eveningness scores indicate morningness. IQR = Interquartile Range.

* p < .05

** p < .01

In all models, age and gender were included as covariates since previous studies report that they are associated with chronotype [38] and satisfaction with life [39] as well as with religiosity [40, 41].

A post hoc power analysis using GPower software found the sample was sufficiently large to detect a small effect size at 80% power for a medium-magnitude Pearson correlation coefficient (r = 0.30). According to previous studies investigating the association between morningness-eveningness, religiosity, conscientiousness, and satisfaction with life (e.g., [11, 12]), the final sample size is adequate for replicating the previously obtained effects, taking into account their magnitude, and, in consequence, enabling the reliable test of the proposed model.

All statistical analyses were conducted using IBM SPSS 26.0.0.1 for Windows, along with Hayes’ [42] PROCESS macro version 3.5 for mediation analyses (Model 6).

### Results

#### Correlation analyses

**Fig 1:**
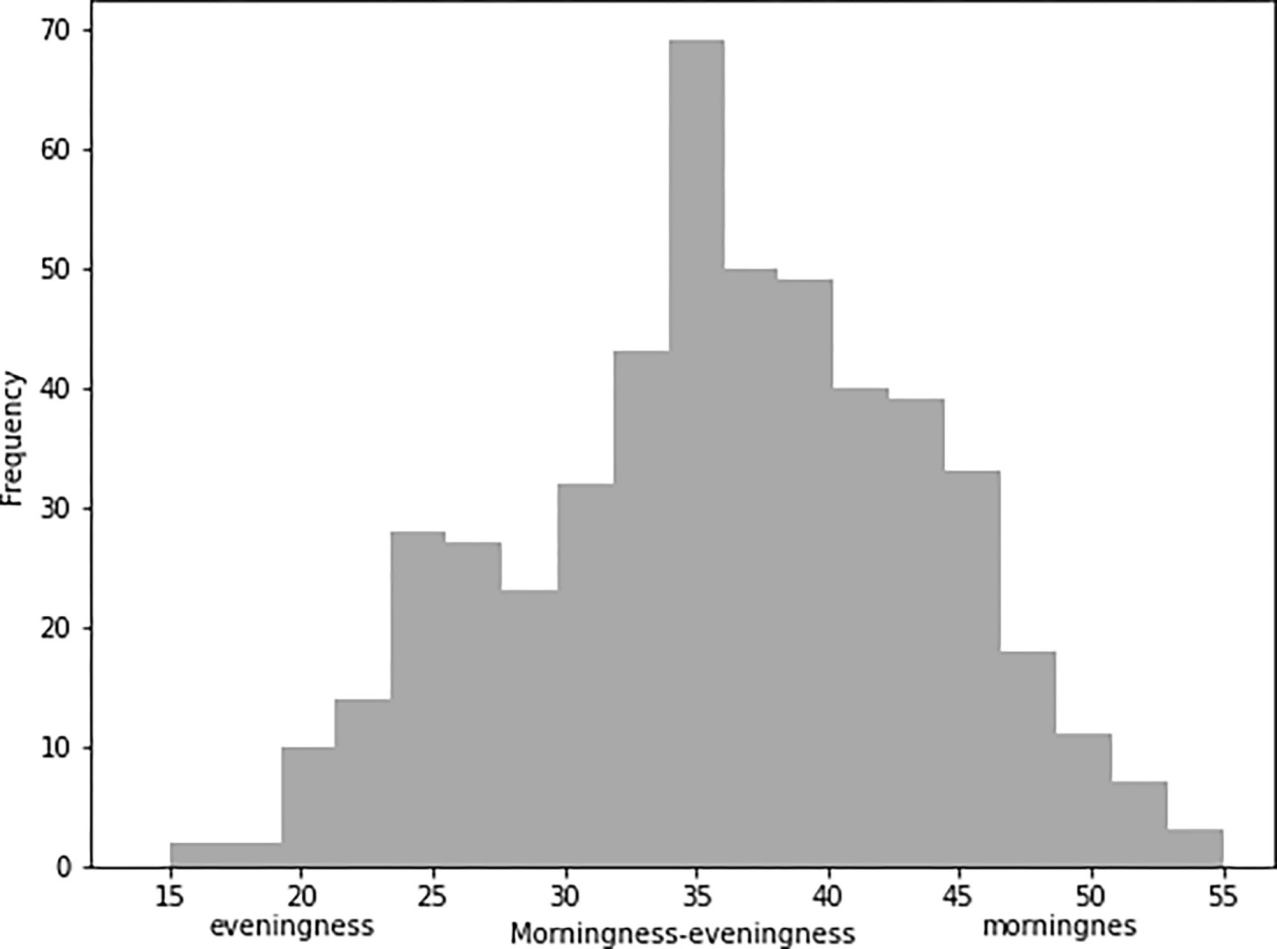
Distribution of morningness-eveningness (CSM scores) in the present sample (Study 1).

The matrix of partial correlations between the measured variables, as well as the reliability of the psychometric measures and descriptive statistics (means, standard deviations, skewness, kurtosis, and interquartile range), are provided in Table 1. The distribution of morningness-eveningness (CSM scores) in the present sample is provided in Fig 1.

Morningness was positively related to conscientiousness. Moreover, morningness was positively associated with both satisfaction with life and belief in God. Belief in God correlated positively with both life satisfaction and conscientiousness. Finally, satisfaction with life positively correlated with conscientiousness.

#### Mediation analyses

We conducted a mediation analysis to verify our main hypothesis that the association between morningness-eveningness and satisfaction with life is mediated by belief in God and verify whether this relationship may be mediated by conscientiousness. Age and gender were introduced to the model as control variables.

**Fig 2:**
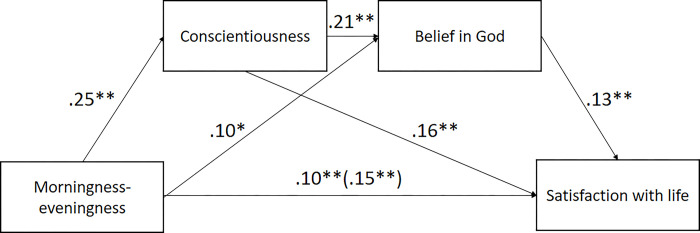
Mediation of the association between morningness-eveningness and satisfaction with life by conscientiousness and belief in God (controlling for gender and age). *p < 0.05; **p < 0.01.

As shown in Fig 2, both conscientiousness and belief in God were significant mediators of the association between morningness-eveningness and satisfaction with life. The total indirect effect amounted to .05 (95% confidence intervals: .03–.08). The individual mediator effects were as follows: .01 for belief in God (95% CI: .00–.03) and .04 for conscientiousness (95% CI: .01–.06). The ratio of indirect to total effect amounted to .33; thus, approximately 33% of the effect may be explained by belief in God and conscientiousness.

### Discussion

In Study 1, we found that morningness was related to a higher conscientiousness and belief in God. We also found that the link between morningness-eveningness and life satisfaction was partly mediated by belief in God, which relationship was, in turn, partly mediated by conscientiousness such that higher levels of conscientiousness and belief in God partly explained the association between morningness and satisfaction with life.

Our results show that belief in God may partially underpin the relationship between morningness-eveningness and satisfaction with life. Belief in God may thus be especially important for evening-oriented individuals who tend to report a lower level of life satisfaction.

Even though Study 1 was conducted on a substantial number of participants, there are at least two important limitations that should be acknowledged. In this study, we used a 3-item scale to measure belief in God which in our study had rather limited reliability (Cronbach’s alpha = .64). Therefore, since this measure turned out to be suboptimal, in Study 2 we decided to both replicate and extend our preliminary findings using a more sophisticated measure of religiosity, which includes different aspects of religious life and therefore provides a comprehensive and reliable estimate of one’s attitude toward religion. Moreover, in Study 1 we did not take into account participants’ religious affiliations. Therefore, in study 2 we decided to control for denominational differences in order to exclude their eventual influences on the obtained effects.

## Study 2

### Method

#### Participants

A total of 774 participants (390 women and 384 men) aged 18 to 36 years (M = 28.26, SD = 4.98) took part in the second study. They were recruited using random sampling by a professional company specializing in panel research and completed questionnaires described below through the Qualtrics platform. Over half of the participants (47%) had university degrees, 43% had secondary education, 7% had vocational education, 2% had lower secondary education, and above 1% had primary education. All of the participants declared that Poland was their country of origin. In terms of religious affiliation, about 74% of the participants identified as Roman Catholics, below 1% as Greek Catholics, below 1% as Orthodox Christians, 1% as Protestants, below 1% as Old Catholics, below 1% as Jehovah’s Witnesses, 3% of participants described their belief as "Other", and 20% identified as nonbelievers.

All participants gave written informed consent before participating in the study. The research was conducted in line with the ethical standards of the Declaration of Helsinki, and the procedure was accepted by the Research Ethics Committee at the Faculty of Psychogy, University of Warsaw.

#### Measures

Chronotype, personality traits, and life satisfaction were measured as described above in Study 1. Instead of the previously used measure of belief in God, in Study 2 we included a well-established measure of religiosity.

*Religiosity* conceptualized as the importance of religious meanings in personality was assessed using the Polish version of the Centrality of Religiosity Scale [43, 44]. The scale consists of 15 items spanning five core dimensions of religiosity, i.e., intellect (e.g., “How interested are you in learning more about religious topics?”); ideology (e.g., “To what extent do you believe that God or something divine exists?”); public practice (e.g., “How often do you take part in religious services?”); private practice (e.g., “How often do you pray?”); and religious experience (e.g., “How often do you experience situations in which you have the feeling that God or something divine is present?). The majority of items within the scale used a five-point response scale from “not at all” or “never” to “very much so” or “very often”. For the two questions regarding religious practices objective frequencies from “never” to either “more than once a week” (religious services) or “several times a day” (prayer) were recorded and then transformed into one of five levels according to the original instructions by [43]. The overall score in religiosity was calculated as a sum of all items from all five subscales, thus ranging from 15 to 75.

### Statistical analyses

The statistical analysis was performed in the same way as described above in Study 1. Having a vast majority of Roman Catholics within our sample and taking into account that in Study 1 non-believers are also included, we decided to run a mediation analysis on these two subsamples in order to reduce potential confounding variability resulting from having a small proportion of other believers. Therefore, a total of 728 individuals participated in the study (570 Catholics and 158 non-believers).

### Results

#### Correlation analyses

**Fig 3:**
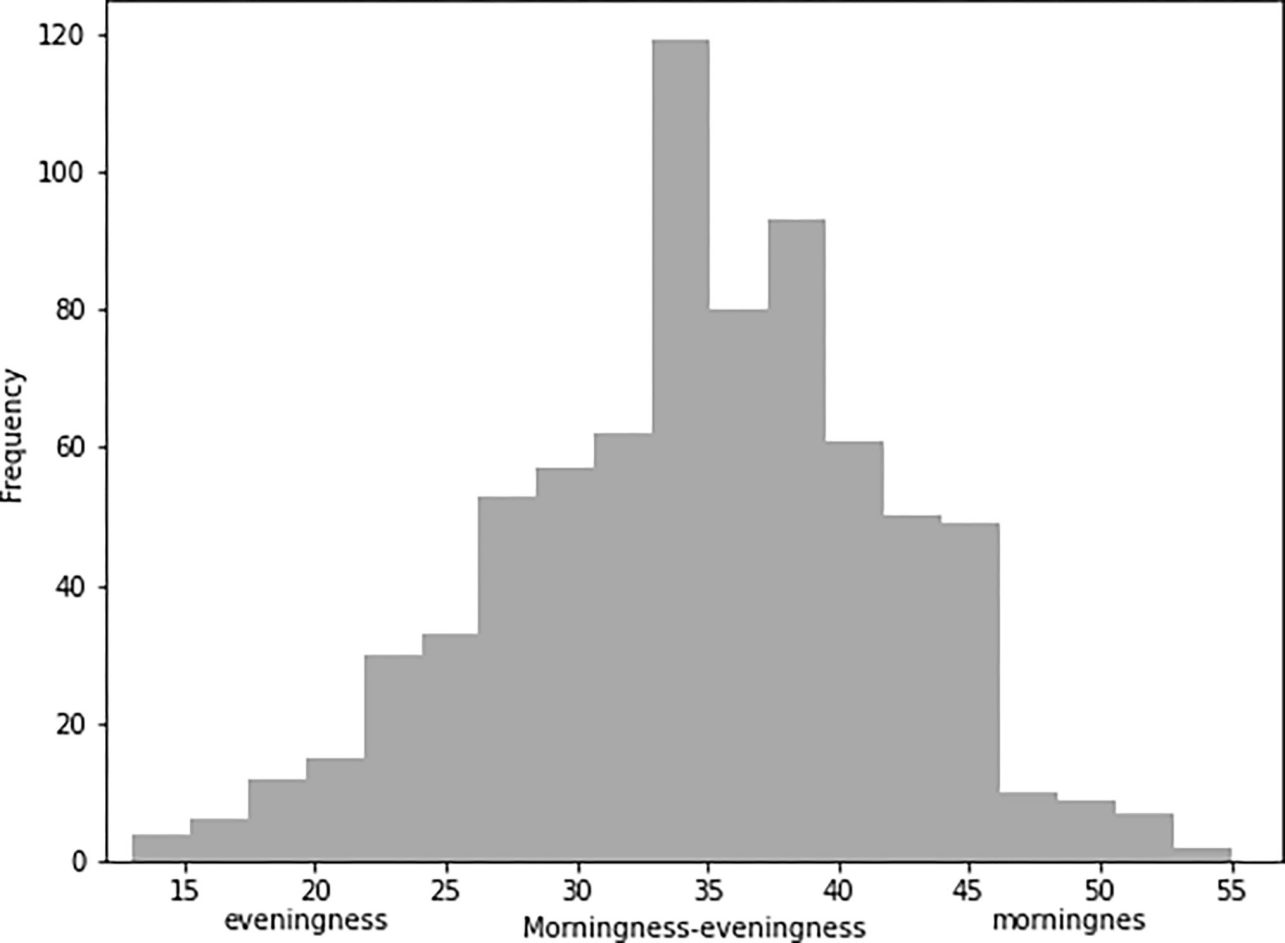
Distribution of morningness-eveningness (CSM scores) in the present sample (Study 2).

The matrix of partial correlations between the measured variables, as well as the reliability of the psychometric measures and descriptive statistics (means, standard deviations, skewness, kurtosis, and interquartile range), are provided in Table 2. The distribution of morningness-eveningness (CSM scores) in the present sample is provided in Fig 3.

**Table 2:** Means, standard deviations, skewness, kurtosis, interquartile range, Cronbach’s alphas, and bivariate correlations between variables included in the study 2 (N = 728).

	M	SD	Skewness	Kurtosis	IQR	α	1.	2.	3.	4.	5.	6.	7.	8.	9.
1. Morningness-eveningness	34.67	7.26	-.23	-.11	10	.88	-								
2. Ideology	9.48	4.16	-.19	-1.27	7	.94	.11*	-							
3. Private practice	7.61	4.01	.38	-1.23	7	.91	.17**	.82**	-						
4. Religious experience	6.41	3.09	.58	-.52	6	.90	.12**	.77**	.82**	-					
5. Intellect	6.63	2.76	.60	-.24	5	.86	.11*	.66**	.74**	.75**	-				
6. Public practice	7.36	3.89	.52	-1.06	7	.88	.19**	.77**	.84**	.77**	.73**	-			
7. Overall religiosity	37.49	16.22	.29	-1.06	27	.97	.16**	.91**	.94**	.91**	.84**	.92**	-		
8. Satisfaction with life	3.88	1.35	-.15	-.43	1.80	.92	.23**	.17**	.22**	.19**	.21**	.22**	.22**	-	
9. Conscientiousness	3.65	.63	-.19	-.18	.90	.83	.23**	.15**	.16**	.11*	.04	.12**	.13**		-

Note. High morningness-eveningness scores indicate morningness; IQR = Interquartile Range.

* p < .05

** p < .01

Morningness was positively associated with general religiosity and all its dimensions, as well as with greater satisfaction with life and higher conscientiousness. Religiosity correlated positively with life satisfaction and conscientiousness. Finally, satisfaction with life positively correlated with conscientiousness.

–

#### Mediation analyses

We conducted a mediation analysis in the same way as described above in Study 1.

**Fig 4:**
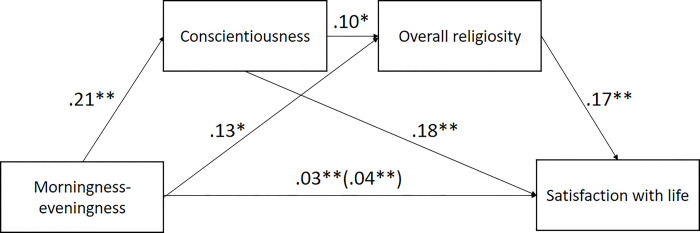
Mediation of the association between morningness-eveningness and satisfaction with life by conscientiousness and overall religiosity (controlling for gender and age). *p < 0.05; **p < 0.01.

As shown in Fig 4, both conscientiousness and religiosity were significant mediators of the association between morningness-eveningness and satisfaction with life. The total indirect effect amounted to .01 (95% confidence intervals: .01–.02). The individual mediator effects were as follows: .01 for religiosity (95% CI: .00–.01) and .01 for conscientiousness (95% CI: .00–.01). The ratio of indirect to total effect amounted to .28; thus, approximately 28% of the effect may be explained by religiosity and conscientiousness.

### Discussion

In Study 2, we conceptually replicated the previous findings, this time using a comprehensive scale of religiosity that consists of several components, including belief in God but also religious practice. As in Study 1, morningness was related to higher conscientiousness. In addition, we found that morningness was positively correlated with all dimensions of religiosity.

Furthermore, we confirmed that the link between morningness-eveningness and life satisfaction is partly mediated by religiosity, which relationship is, in turn, partly mediated by conscientiousness such that higher levels of conscientiousness and religiosity explain the association between morningness and satisfaction with life. Importantly, obtained results also remained significant only in the group of Roman Catholic believers (see Supplementary material).

## General discussion

The aim of the present studies was to simultaneously investigate the role of both conscientiousness and religiosity in the association between morningness-eveningness and satisfaction with life. As expected, across the two studies, we found that morningness was related to higher religiosity and conscientiousness. We also found that the positive link between morningness and life satisfaction was partly mediated by religiosity, which relationship proved, in turn, partly mediated by conscientiousness.

The finding that conscientiousness contributes to higher life satisfaction among morning-oriented individuals is consistent with previous research on the links between chronotype and this personality trait [45]. Conscientiousness typically demonstrates beneficial associations of small-to-medium magnitude with life satisfaction, positive affect, and lack of negative affect [14]. Moreover, higher conscientiousness may also act as a protective factor against higher depressive symptoms among evening-oriented individuals [5]. However, an important novel result from the current research is that the impact of larks’ conscientiousness on well-being [13] is not necessarily direct but may also work through its association with religiosity. Namely, in line with other investigations, we suggest that higher conscientiousness can foster the development of religiosity [16] and in consequence, indirectly, contribute to the formation of attitudes that have favorable psychological outcomes [46, 47]. As classically put forward by Gordon Allport [48] in his expressiveness perspective, personality traits can stimulate people to develop specific cultural adaptations that allow them to fulfill their basic tendencies. Conscientious individuals, therefore, can seek religious environments, in which they are able to express their personality because acting upon their personal tendencies feels good and brings satisfaction [48, 49].

Importantly, although the link between morningness and religiosity was mediated via conscientiousness, we established that there was also a significant direct path from morningness to religiosity (as a mediator contributing to satisfaction with life). It means that at least some portion of the relationship between morningness, religiosity, and life satisfaction could be explained by factors other than elevated conscientiousness. Here, we propose that yet another underlying mechanism may be connected with the level of perceived social support, which is strongly and positively related to life satisfaction [50]. Research indicates that the positive association between morningness and well-being might stem from the higher levels of social support received by morning-oriented individuals [7]. Moreover, the fact that morningness is associated with higher overall perceived social support has been confirmed in other studies as well [51]. This pattern of findings is highly relevant in terms of religiosity because religious believers routinely report larger social networks and more contact with social members [52], which may help them to elevate the social support they receive and perceive [53]. Therefore, we argue that the benefits of social support among morning-oriented individuals may be at least partly derived from active participation in religious groups and communal rituals [47].

Finally, research also indicates that the relationship between morningness-eveningness and satisfaction with life may be associated with sleep parameters and that poor sleep quality and short sleep duration may translate into negative psychological functioning mainly among evening-oriented individuals [54]. Worse sleep quality and insufficient sleep duration have a significant impact on well-being [55, 56]. These sleep deficiencies may be related to the fact that evening-oriented individuals often experience misalignment between their biological clock and social clock (they often do not fit into conventional social and working schedules). Here, religiosity can again play an influential role in regulating sleep timing and promoting healthy sleep habits [23]. For instance, certain religious practices, like the Islamic call for prayer or Advent dawn mass in Catholic churches, require activity at prescribed times during the day including those very early in the morning. Furthermore, religious teachings commonly provide a well-defined structure of weekly, monthly, and yearly observances, which can facilitate personal time management and therefore contribute to a satisfactory pattern of psychosocial [21, 57]. Lastly, religious cognitions such as secure attachment to God or belief in salvation were found to serve as a buffer against the detrimental effects of stress on sleep quality [58]. All of these regulatory features of religiosity can result in morning-oriented individuals experiencing better well-being and enjoying significant life satisfaction benefits compared to evening-oriented individuals.

It is important to note that in our studies, we explored one of the potentially possible mediation models. Based on the research indicating that morningness-eveningness and conscientiousness have a strong biological basis [59, 60], we decided that these factors will be the basis of the studied mediation model. Moreover, our previous studies of causal effects between chronotype and conscientiousness strengthen this hypothesis, indicating the possibility of their common biological basis [61]. Finally, research indicates that religiosity has a strong cultural basis [62]. However, there is a need for further research testing alternative mediation models, including other potentially vital explanatory mechanisms. Moreover, future longitudinal studies might shed more light on the causality in the interplay between morningness-eveningness, conscientiousness, religiosity, and satisfaction with life.

## Limitations & future directions

Although the current research established a robust link between chronotype, conscientiousness, religiosity, and life satisfaction, it is not free from certain limitations. First of all, both current studies were cross-sectional, and therefore any inferences regarding the direction of causality between the investigated variables remain speculative or, at best, are based on previous research. Second, the assessment of all variables in the study was based solely on self-report information and thus potentially subject to a range of biases, including social desirability bias [58]. The unavailability of sociodemographic information (such as marital status, number of children, work status, and schedules), and the inability to statistically control for such effects, may be another limitation of the studies. Moreover, while in this investigation, we focused on either a general belief in God or the overall importance of religion in one’s personality, future studies may extend the present findings by including more specific types of religiosity, such as religious orientations [63] or religious fundamentalism [64]. Similarly, the current findings should be replicated with participants of other cultural backgrounds, as prior work suggests substantial cross-cultural differences in both understanding and use of time [65] as well as religiosity and its relationship with other psychological factors [49]. Moreover, a potential limitation of the research may also be the strength of the obtained correlation effects between the variables in the studies. However, the correlations were statistically significant and are consistent with previous studies [5, 6, 15]. Also, in Study 1, we used a 3-item scale to measure belief in God which in our study had rather limited reliability (Cronbach’s alpha = .64). However, the same scale has been previously used in several empirical investigations [30, 33] and shows good validity and strong correlations with other religiosity scales [33]. In future studies, diurnal typology could be extended using other measurements, such as the Munich Chronotype Questionnaire (MCTQ) [66] or the three-dimensional Morningness-Eveningness-Stability-Scale Improved (MESSi) model [67], which could provide a more detailed picture of investigated relationships. Furthermore, a longitudinal study could provide a stronger test of our hypotheses and confirm the stability of obtained effects.

## Conclusion

In conclusion, our current results show that the link between morningness-eveningness and life satisfaction is partly mediated by religiosity, which, in turn, is partly mediated by conscientiousness. It means that more morning-oriented individuals may benefit from higher psychological well-being thanks to both personality characteristics and attitudes towards religion. That is to say, larks tend to be more conscientious, which may render them more inclined toward religiosity, whereas their elevated religiosity can substantially contribute to overall satisfaction with life.

## Supporting information

S1 Fig Mediation of the association between morningness-eveningness and satisfaction with life by conscientiousness and overall religiosity in the group of Roman Catholic believers (controlling for gender and age). *p < 0.05; **p < 0.01.
(PNG)
