# Supplementary figures and images for: Godless owls, devout larks: Religiosity and conscientiousness are associated with morning preference and (partly) explain its effects on life satisfaction
Source: PLoS One. 2023 May 24;18(5):e0284787. doi: 10.1371/journal.pone.0284787 (PMC10208493; doi:10.1371/journal.pone.0284787)

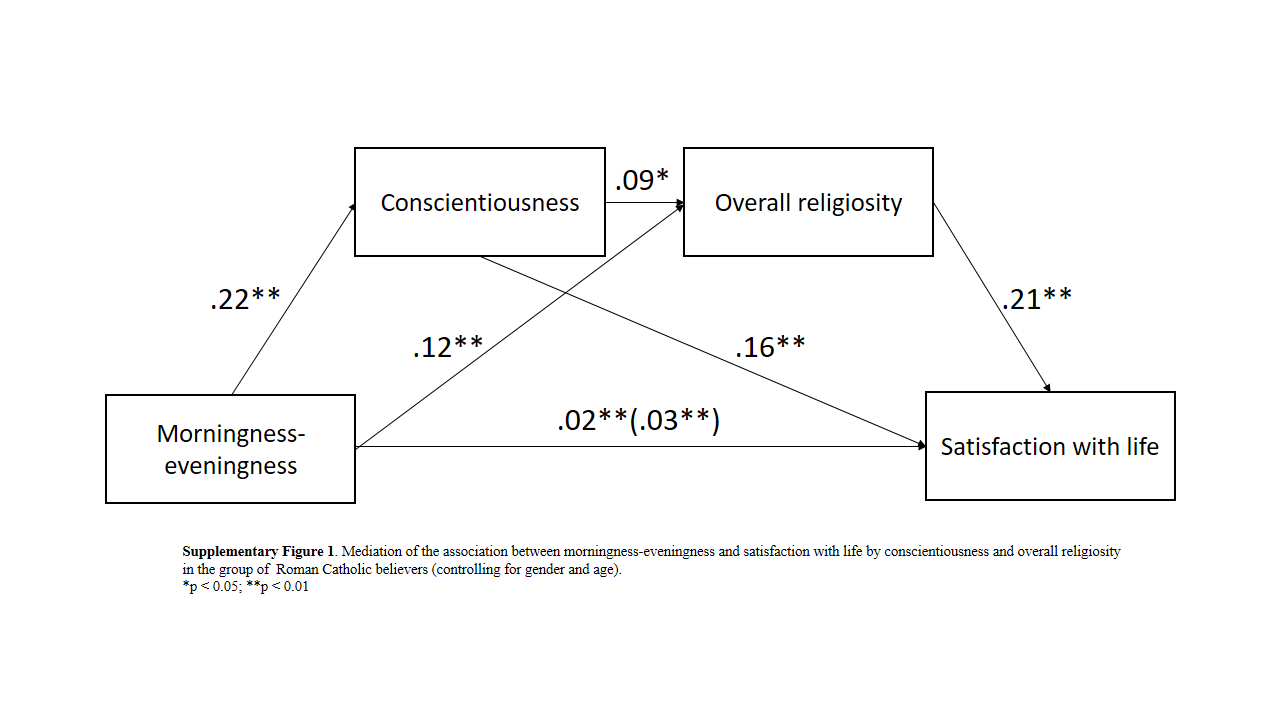

Supplement: S1 Fig — *p < 0.05; **p < 0.01. (PNG) [file pone.0284787.s001.png]
